# Genotyping of *Acinetobacter baumannii* isolates from a tertiary care hospital in Cochin, South India

**DOI:** 10.1099/acmi.0.000662.v4

**Published:** 2023-11-28

**Authors:** Vineeth Rajan, Ardhra Vijayan, Ambili Puthiyottu Methal, Justus Lancy, Gopalan Krishnan Sivaraman

**Affiliations:** ^1^​ Microbiology, Fermentation and Biotechnology Division, ICAR-Central Institute of Fisheries Technology, Cochin, India; ^2^​ Department of Microbiology, Government Medical College, Ernakulam, India

**Keywords:** *Acinetobacter baumannii*, carbapenem resistance, disinfectant resistance, biofilm formation, pulsed-field gel electrophoresis

## Abstract

*
Acinetobacter baumannii* poses a significant challenge in healthcare settings across the globe, with isolates exhibiting carbapenem resistance at unprecedented rates. Here, we characterized a collection of *A. baumannii* isolates (*n*=64) recovered during the period September 2020 – November 2021 at a teaching hospital in Cochin, South India. The species identity of the isolates was confirmed with *bla*_OXA-51-like_ PCR. The major carbapenemase determinants identified were *bla*_OXA-23-like_ (45, 70.3 %) and *bla*_NDM-1_ (31, 48.4 %); co-occurrence of these genes was also observed in 27 (42.2 %) isolates. Other resistance genes identified included *bla*_PER_ (34, 53.1 %), *aac(6')-Ib-cr* (42, 65.6 %), *qnrS* (25, 39.1 %), *sul1* (32, 50 %), *sul2* (33, 51.6 %), *strA/strB* (36, 56.3 %), *aphA1-Iab* (35, 54.7 %) and *tetB* (32, 50 %). Mapping PCR revealed the insertion element, IS*AbaI* upstream of *bla*_OXA-23-like_ in all isolates possessing this gene. Concerning disinfectant resistance, all isolates carried the quaternary ammonium compound (QAC) resistance gene, *qacEΔ1*. Minimal inhibitory concentration (MIC) of benzalkonium chloride was high among the isolates and ranged from 8 to 128 µg ml^−1^. However, low MICs were observed for chlorhexidine and triclosan, with the majority (54, 80.6 %) of isolates showing an MIC of 2 µg ml^−1^ for chlorhexidine and all isolates exhibiting MICs of ≤0.125 µg ml^−1^ for triclosan. Further, all isolates were strong biofilm-producers, as assessed by the crystal violet-based microtitre plate assay. The *Apa*I-pulsed-field gel electrophoresis (PFGE) revealed the multi-clonal nature of the isolates, with 16 clusters and 16 unique pulsotypes identified at a cut-off of 80 %. In short, this study provides useful data on the molecular features of *A. baumannii* from this region, which could be helpful to assess the local epidemiology of this pathogen and also to devise infection control strategies.

## Data Summary

The authors confirm all supporting data, code and protocols have been provided within the article or through supplementary data files.

## Introduction

*Acinetobacter baumannii* has emerged as an important nosocomial pathogen that mainly infects critically ill patients. However, cases of community-acquired infections which are usually associated with preexisting conditions such as old age, diabetes, cancer, obstructive pulmonary disorders, alcoholism etc. have also been reported [1]. The Centres for Disease Control and Prevention (CDC) has included carbapenem-resistant *Acinetobacter* in the category of ‘urgent threat’, thus calling for increased surveillance and prevention activities to manage this pathogen [2]. Infections caused by *A. baumannii* include pneumonia, bacteremia, urinary tract infections, meningitis, skin/wound infections etc. Closely related and phenotypically indistinguishable species of *Acinetobacter* have been clustered into what is called *Acinetobacter calcoaceticus-baumannii* (Acb) complex. The Acb complex comprises five pathogenic species namely *A. baumannii *, *A. nosocomialis *, *A. pittii *, *A. seifertii* and *A. dijkshoorniae* as well as the non-pathogenic *A. calcoaceticus *. The epidemiological success of *A. baumannii* is attributed mainly to its ability to persist in the environment and to acquire resistance to various drugs including carbapenems. Resistance to desiccation, disinfection and oxidative stress, as well as the ability to form biofilms are the hallmarks of this pathogen that enable it to thrive successfully in the apparently harsh healthcare environments [3].

*Acinetobacter baumannii* has acquired resistance to virtually all antibiotics including fluoroquinolones, cephalosporins, aminoglycosides and carbapenems. Incidence of carbapenem-resistant *A. baumannii* (CRAB) has increased alarmingly in the past decade, threatening one of the last resort treatment options for this pathogen. A recent multicentre surveillance study from India documented carbapenem susceptibility rate as low as ~12 % for *A. baumannii* isolates [4]. Intense usage of carbapenems has been shown to facilitate the persistence of CRAB isolates in intensive care units, causing sustained outbreaks [5]. Various intrinsic and acquired mechanisms have been known to confer carbapenem resistance to *A. baumanni* which include production of carbapenemases, increased expression of efflux pumps, alterations in porins and modifications in penicillin-binding proteins (PBPs) [6]. Genes encoding diverse carbapenem-hydrolysing β-lactamases belonging to class A (*bla*_KPC_ and *bla*_GES_), class B (*bla*_NDM_, *bla*_SIM_, *bla*_IMP_ and *bla*_VIM_) and class D (*bla*_OXA-23-like_, *bla*_OXA-24-like_, *bla*_OXA-40-like_, *bla*_OXA-51-like_, *bla*_OXA-58-like_ and *bla*_OXA-143-like_) have been reported in *A. baumannii* [7, 8]. However, class D enzymes such as OXA-23 have been by far the most common carbapenemases among CRAB isolates from many countries including India [9–11]. The intrinsic *bla*_OXA-51_ gene codes for a weak carbapenemase and is often employed as a marker for the species identification of *A. baumannii* [7]. The expression and mobilization of OXA enzymes are known to be regulated by certain insertion sequences (IS) such as the IS*AbaI* associated with *bla*_OXA_ genes in *Acinetobacter* [6]. Owing to the increased prevalence of carbapenem resistance, limited treatment options are available for managing severe CRAB infections. This mainly includes mono- and combination therapy involving colistin, tigecycline, carbapenem, rifampicin, amikacin, sulbactam and minocycline. However, toxicity, suboptimal pharmacokinetics and/or increasing resistance, associated with one or more of these agents pose significant challenges to effectively managing the infection.

Understanding the molecular epidemiology of *Acinetobacter* is vital to monitor its spread and devise containment strategies in hospitals and long term care facilities. In this study, we characterized a collection of isolates of *A. baumannii* from a tertiary care hospital in the city of Cochin, India with respect to their antibiotic resistance features and genetic diversity.

## Methods

### Study setting and isolates

This study analysed a total of 64 isolates of *A. baumannii* collected as part of the routine clinical testing at the Government Medical College, Ernakulam, India during the period September 2020–November 2021. The study was approved by the Institutional Ethical Committee of Government Medical College Ernakulam vide ref. no. IEC40/2020 dated 16 November 2020. The isolates were recovered from cases of pneumonia (*n*=17), urinary tract infection (UTI) (*n*=16), surgical site infection (*n*=7), diabetic foot (*n*=7), sepsis (*n*=7), COPD exacerbation (*n*=2), abscess (*n*=2), pleural effusion (*n*=1), biliary peritonitis (*n*=1), cellulitis (*n*=1), burn infection (*n*=1), gangrene (*n*=1) and pyoderma (*n*=1). The isolates were identified as *A. baumannii* by BD Phoenix M50 automated system (BD Diagnostics, USA) and later confirmed with a PCR for *bla*_OXA-51-like_ gene, which is intrinsic to this species [12].

### Antibiotic susceptibility testing

Phenotypic susceptibility of the isolates towards selected antibiotics (amikacin, cefepime, ceftriaxone, ceftazidime, ciprofloxacin, gentamicin, imipenem, levofloxacin, meropenem, piperacillin-tazobactam, tetracycline and trimethoprim-sulfamethoxazole) was determined using the ID/AST combo panel, NMIC/ID55 in BD Phoenix M5O automated system. The panel consists of an ID side containing wells with dried substrates for bacterial identification and an AST side containing wells having varying concentrations of antibiotics. Briefly, bacterial colonies from pure cultures (grown on trypticase soy agar- TSA) were transferred to the BD Phoenix ID broth and the inoculum density was adjusted to 0.5 McFarland using BD PhoenixSpec nephelometer. Twenty five microlitres of the adjusted ID broth suspension was then transferred to the BD Phoenix AST broth with the AST indicator which is a resazurin-based dye. The suspensions (ID broth inoculum and AST broth inoculum) were then poured to the corresponding fill ports in the panel. Panels were sealed and loaded into the instrument for incubation at 35 °C for around 16 h. BD Phoenix system is connected to the data management software BD EpiCenter (version V7.22/V6.41A) which analyses test results and generates reports. EpiCenter implements CLSI breakpoints and provides sensitive/resistant/intermediate (S/I/R) interpretations based on the MIC of the tested antibiotics.

### PCR screening for antibiotic resistance genes

Genomic DNA was extracted from all the cultures using DNeasy Blood and Tissue kit (Qiagen, Germany) following the manufacturer’s instructions and used for subsequent PCR analyses. All PCRs were carried out in a final volume of 25 µl containing 1X Jumpstart RedTaq Ready Mix (Sigma-Aldrich, USA), primers in the required concentrations and 2 µl of the extracted DNA. Screening for the four groups of *bla*_OXA_ carbapenemase genes (*bla*_OXA-23-like_, *bla*_OXA-24-like_, *bla*_OXA-51-like_ and *bla*_OXA-58-like_) was performed using a previously described multiplex PCR [12]. The isolates were also screened for other carbapenemase genes (*bla*_KPC_, *bla*_IMP_, *bla*_VIM_, *bla*_SIM-1_ and *bla*_NDM-1_), various extended-spectrum β-lactamase (ESBL) genes (*bla*_TEM_, *bla*_SHV_, *bla*_CTX-M_ and *bla*_PER_), fluoroquinolone resistance genes (*qnrA, qnrB, qnrS, aac(6')-Ib-cr, qepA, oqxA* and *oqxB*), aminoglycoside resistance genes (*strA, strB,* and *aphA1-Iab*), sulphonamide resistance genes (*sul1* and *sul2*) and tetracycline resistance genes (*tetA* and *tetB*) using primers and PCR conditions described elsewhere [13–17] (Table S1, available in the online version of this article).

### Detection and mapping the position of IS*AbaI*

Presence of the insertion sequence, IS*AbaI* was investigated in all the isolates as described previously [18]. In order to map the position of IS*AbaI* in relation to *bla*_OXA-51_ and *bla*_OXA-23_ genes, two independent PCRs were carried out using the following combinations of primer pairs: (a) IS*AbaI*-forward primer (IS*AbaI*F) and *bla*_OXA-51-like_ reverse primer (OXA-51-likeR); and (b) IS*AbaI*F and *bla*_OXA-23-like_ reverse primer (OXA-23-likeR) [19].

### Determination of MICs of disinfectants

Minimal inhibitory concentrations (MICs) of three disinfectants namely benzalkonium chloride, chlorhexidine and triclosan were determined using agar dilution method according to CLSI guidelines [20]. Stock solutions of 5120 µg ml^−1^ of the disinfectants (all purchased from Sigma, USA) were prepared in appropriate solvents: benzalkonium choloride in water, chlorhexidine in dimethyl formamide and triclosan in ethanol. Suspensions of pure bacterial cultures were prepared in 3 ml of 0.85 % saline by using overnight colonies from TSA plates, and the preparation was adjusted to a turbidity equivalent to that of 0.5 McFarland standard. The suspensions were further ten-fold diluted using 0.85 % saline, and 2 µl (an inoculum of ~10^4^ c.f.u. per spot) was spotted on Mueller-Hinton agar plates containing disinfectants at various concentrations ranging from 0.125 to 512 µg ml^−1^. The reference strain *Escherichia coli* ATCC25922 was used as quality control. Plates were incubated at 37 °C for 24 h and MIC was recorded as the lowest concentration of the disinfectant that completely inhibited bacterial growth.

### Screening for disinfectant resistance genes

All the isolates were screened for disinfectant resistance genes such as *qacE, qacEΔ1, qacG* and *qacH* using previously described PCRs [21].

### Screening for biofilm formation

Biofilm-forming ability of the isolates was assessed using crystal violet assay as previously described by Naves *et al.* with slight modifications [22]. Briefly, 20 µl of the each overnight culture of *A. baumannii* was inoculated to 96-well microtitre plate containing 180 µl of brain heart infusion broth with 2 % glucose. The experiment was performed in triplicates and uninoculated wells served as negative control. The plate was incubated at 37 °C for 24 h without agitation. After incubation, the medium was carefully removed and the wells were rinsed with 0.85 % saline to remove the unbound bacteria. Methanol was added to the well and incubated for 20 min to fix the biofilm. Plate was then air-dried, 200 µl of 0.1 % crystal violet solution was added to the wells and incubated for 20 min. The residual dye was removed and the wells were rinsed with distilled water. After drying, the bound crystal violet was re-solubilized in 200 µl of 33 % (v/v) glacial acetic acid for 15 min. The optical density was measured at 585 nm using the microplate reader. The biofilm-forming ability was evaluated by comparing the average OD value (OD_T_) for each tested strain with the cut-off OD (OD_C_), which is the average OD of the negative control plus three times the standard deviation of the ODs of negative control wells. Isolates with OD_T_ >4 OD_C_ were considered as strong biofilm-producers.

### Molecular subtyping by pulsed-field gel electrophoresis

The genetic relatedness among the isolates was assessed by *ApaI*-pulsed-field gel electrophoresis (PFGE) [23]. Pure cultures of *A. baumannii* were inoculated on TSA plates and incubated at 37 °C overnight. A loopful of the bacteria was taken from the agar surface and suspended in 3 ml of cell suspension buffer (100 mM Tris, 100 mM EDTA, pH 8). Cell suspensions were adjusted to an OD_610_ of 0.8–1 (~10^9^ cells ml^−1^). Two hundred microlitres of the cell suspensions were mixed with 10 µl of proteinase K (20 mg ml^−1^) in 1.5 ml microcentrifuge tubes. It was then mixed with 200 µl of molten megabase agarose (Bio-Rad, Germany) prepared in TE buffer (10 mM Tris, 1 mM EDTA, pH 8). The agarose-cell suspension was immediately dispensed into the wells of reusable plug moulds (Bio-Rad, Germany) and the plugs were allowed to solidify for 10 min. Plugs were then transferred to a 50 ml polypropylene tubes containing 5 ml cell lysis buffer (50 mM Tris, 50 mM EDTA, 1 % sarcosine, pH 8) and 25 µl of proteinase K (20 mg ml^−1^). Lysis was carried out at 55 °C in a shaking incubator for 2 h with vigorous agitation (180 r.p.m.). After lysis, buffer was removed carefully and the plugs were washed twice in sterile distilled water and thrice in TE buffer at 55 °C in a shaking incubator. Plugs were stored in fresh TE buffer at 4 °C until use.

A slice from each plug was cut using a sterile coverslip and subjected to a pre-digestion incubation in 200 µl 1X restriction buffer (NEB, Germany) for 15 min at 25 °C . This restriction buffer was replaced with 200 µl of fresh restriction buffer containing 30 U of *ApaI* (NEB, Germany) and 100 µg ml^−1^ bovine serum albumin (BSA). The tubes were incubated at 25 °C for 2 h. The restriction mixture was then replaced with 200 µl of 0.5 X TBE buffer (10X TBE has 0.9 M Tris, 0.9 M boric acid and 20 mM EDTA, pH 8.3). Plugs were loaded into 1 % agarose gel (Megabase agarose, Bio-Rad) prepared in 0.5 X TBE buffer. Plugs of Salmonella serotype Braenderup H9812 digested with *XbaI* (50 U per plug, NEB) were used as molecular markers. Electrophoresis was performed in a CHEF-Mapper XA system (Bio-Rad, United States) with 0.5 X TBE as running buffer. Electrophoretic conditions included a voltage of 6 V cm^−1^, pulse time ranging from 5 to 20 s, an included angle of 120° and a total run time of 19 h. The gels were stained in ethidium bromide (1 µg ml^−1^) and visualized under UV illumination. Gel images were exported to Bionumerics software package 7.6.3 (Applied Maths, Belgium) and cluster analysis was performed by Unweighted Pair Group Method with Arithmetic mean (UPGMA) and Dice coefficient with a tolerance setting of 1.5 %. Genetic similarity of the isolates was assessed at a cut-off of 80 %.

## Results

### Antibiotic susceptibility of the isolates

The identity of the isolates (*n*=64) as *A. baumannii* was confirmed by a positive PCR for *bla*_OXA-51-like_ gene. Percentage of resistance among the isolates towards different antibiotics was as follows: amikacin (41, 64.1 %), cefepime (45, 70.3 %), ceftriaxone (55, 85.9 %), ceftazidime (45, 70.3 %), ciprofloxacin (43, 67.2 %), gentamicin (39, 60.9 %), imipenem (38, 59.4 %), levofloxacin (39, 60.9 %), meropenem (40, 62.5 %), piperacillin-tazobactam (43, 67.2 %), tetracycline (38, 59.4 %) and trimethoprim-sulfamethoxazole (37, 57.8 %). Table 1 shows the various resistance patterns observed among the isolates.

**Table 1.** Antibiotic resistance patterns observed among the isolates of *A. baumannii* in the present study

Sl no.	Phenotypic resistance pattern	no. of resistant isolates (%)
1	AK-CPM-CTR-CAZ-CIP-GEN-IP-LE-MP-PTZ-TET-SXT	19 (29.7 %)
2	CTR	8 (12.5 %)
3	AK-CPM-CTR-CAZ-CIP-GEN-IP-LE-MP-PTZ-TET	7 (10.9 %)
4	CPM-CAZ-CIP-GEN-IP-LE-MP-PTZ-SXT	3 (4.7 %)
5	TET	1 (1.6 %)
6	AK-CPM-CTR-CAZ-CIP-GEN-IP-LE-MP-PTZ- SXT	1 (1.6 %)
7	AK-CPM-CTR-CAZ-GEN-LE-PTZ-SXT	1 (1.6 %)
8	AK-CPM-CTR-CAZ-CIP-IP-MP-PTZ	1 (1.6 %)
9	CPM-CTR-CAZ-LE-TET-SXT	1 (1.6 %)
10	AK-CPM-CTR-CAZ-CIP -IP-MP-PTZ-TET-SXT	1 (1.6 %)
11	AK-CTR-CAZ-GEN-SXT	1 (1.6 %)
12	CPM-CTR-CAZ-CIP-GEN	1 (1.6 %)
13	AK-CPM-CTR-CIP-IP-LE-MP-PTZ-TET-SXT	1 (1.6 %)
14	AK-CPM-CTR-CIP-GEN-PTZ	1 (1.6 %)
15	AK-CPM-CTR-CAZ-CIP-IP-LE-MP-PTZ-TET-SXT	1 (1.6 %)
16	CAZ-GEN-MP-SXT	1 (1.6 %)
17	CTR-PTZ-SXT	1 (1.6 %)
18	CPM-CTR	1 (1.6 %)
19	CTR-TET-SXT	1 (1.6 %)
20	AK-CPM-CTR-CAZ-CIP-GEN-IP-MP-PTZ-TET	1 (1.6 %)
21	AK-CTR-CAZ-CIP-GEN-LE-SXT	1 (1.6 %)
22	CTR-TET	1 (1.6 %)
23	AK-CPM-CTR-CAZ-CIP-LE-MP-PTZ-TET-SXT	1 (1.6 %)
24	AK-CPM-CTR-CAZ-CIP-GEN-LE-PTZ-TET-SXT	1 (1.6 %)
25	AK-CPM-CTR-CAZ-GEN-IP-LE-MP-PTZ-TET	1 (1.6 %)
26	CTR-CIP	1 (1.6 %)
27	AK-CPM-CAZ-CIP-GEN-IP-LE-MP-PTZ-SXT	1 (1.6 %)

AK, Amikacin; CAZ, Ceftazidime; CIP, Ciprofloxacin; CPM, Cefepime; CTR, Ceftriaxone; GEN, Gentamicin; IP, Imipenem; LE, Levofloxacin; MP, Meropenem; PTZ, Piperacillin-Tazobactam; SXT, Trimethoprim-Sulfamethoxazole; TE, Tetracycline.

### Prevalence of antibiotic resistance genes

Among the carbapenemase genes screened, the intrinsic *bla*_OXA-51-like_ was present in all the isolates (*n*=64), and *bla*_OXA-23-like_ and *bla*_NDM-1_ were detected in 45 (70.3 %) and 31 (48.4 %) isolates respectively. The ESBL gene, *bla*_PER_ was identified in 34 (53.1 %) isolates. Other resistance genes detected were *qnrS* (25, 39.1 %) and *aac(6')-Ib-cr* (42, 65.6 %) coding for fluoroquinolone resistance; *sul1* (32, 50 %) and *sul2* (33, 51.6 %) conferring resistance to sulphonamides; *strA* (36, 56.3 %), *strB* (36, 56.3 %) and *aphA1-Iab* (35, 54.7 %) conferring resistance to aminoglycosides; and the tetracycline resistance gene, *tetB* (32, 50 %). A master chart detailing the phenotypic and genotypic resistance features of the isolates is provided as Table 2.

**Table 2.**
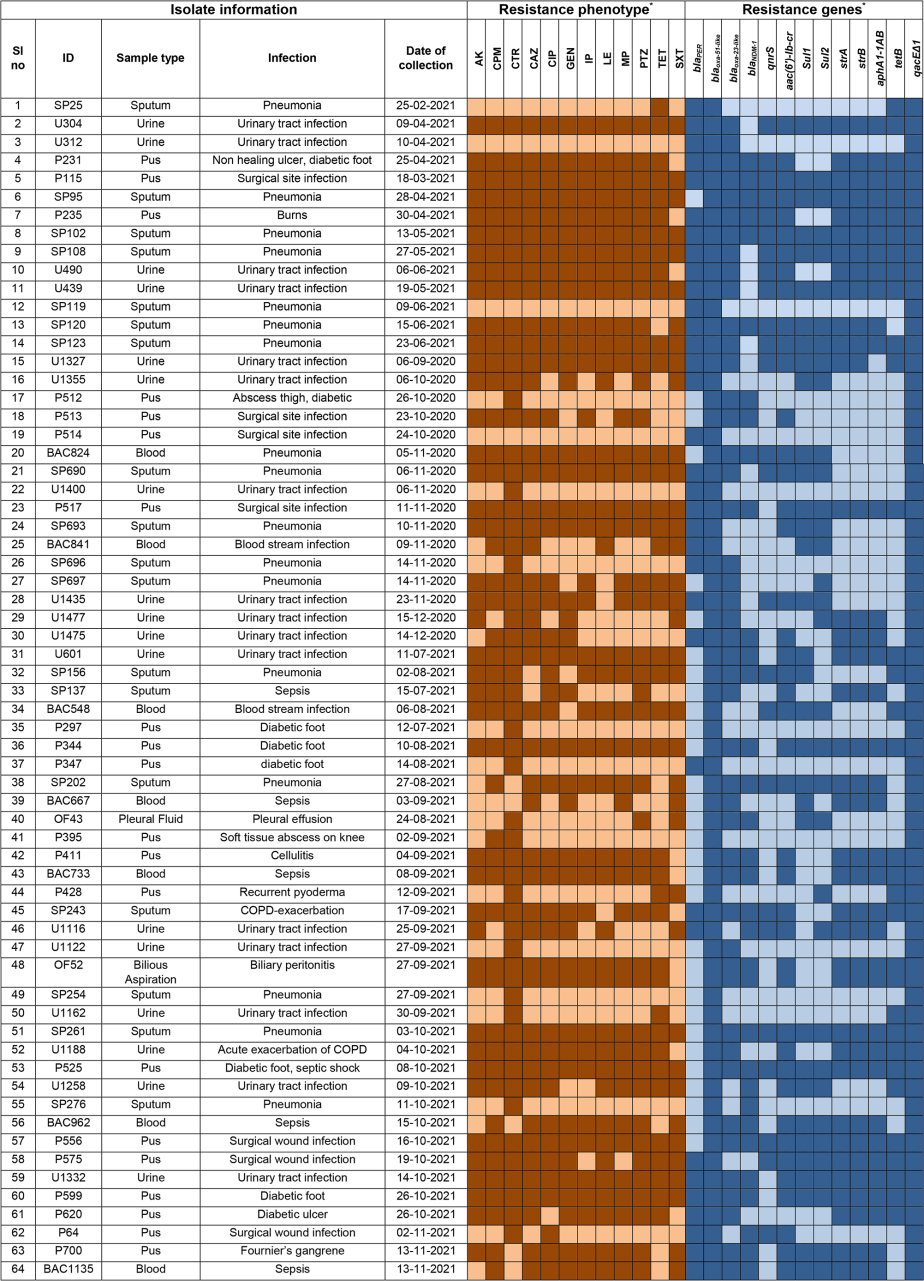
Master chart showing the phenotypic and genotypic features of the isolates

*Darker and lighter shades indicate the presence and absence of the respective resistance phenotype/genes respectively.

AK, Amikacin; CAZ, Ceftazidime; CIP, Ciprofloxacin; CPM, Cefepime; CTR, Ceftriaxone; GEN, Gentamicin; IP, Imipenem; LE, Levofloxacin; MP, Meropenem; PTZ, Piperacillin-Tazobactam; SXT, Trimethoprim-Sulfamethoxazole; TE, Tetracycline.

### IS*AbaI mapping*

All isolates were positive for the insertion element, IS*AbaI*. Mapping PCRs revealed the position of IS*AbaI* relative to *bla*_OXA-23_ and bla*_OXA-51_* genes. All the 45 isolates that were positive for both *bla*_OXA-23_ and *bla*_OXA-51_, gave amplification with a product size of approximately 1.6 kb in a PCR using the forward primer for IS*AbaI* and the reverse primer for bla_OXA-23_. This indicated that, in these isolates, the IS element is associated with, and upstream of *bla*_OXA-23_. Concerning the association of IS*AbaI* with *bla*_OXA-51_, only 23 (51 %) out of the 45 gave a band (of around 1.2 kb) in a PCR involving forward primer for IS*AbaI* and the reverse primer for *bla*_OXA-51_. This revealed the presence of IS*AbaI* upstream of *bla*_OXA-51_ in those isolates. However, none of the isolates with *bla*_OXA-51_ as the sole *bla*_OXA_ gene had IS*AbaI* upstream of it.

### Incidence of disinfectant resistance

In general, MIC of benzalkonium chloride was high and ranged from 8 to 128 µg ml^−1^. Of all isolates, 24 (37.5 %), 18 (28.1 %) and 21 (32.8 %) had MICs of 128, 64 and 32 µg ml^−1^ respectively for benzalkonium chloride; a single isolate had an MIC of 8 µg ml^−1^. In the case of chlorhexidine, the majority (53, 82.8 %) of isolates showed an MIC of 2 µg ml^−1^; whereas, six (9.4 %) and five (7.8 %) isolates had MICs of 1.0 and 0.5 µg ml^−1^ respectively. Invariably, all the isolates exhibited a low MIC (≤0.125 µg ml^−1^) of triclosan. Among the disinfectant resistance genes screened, *qacEΔ1* was the only one detected and was present in all the isolates.

### Biofilm formation

**Fig. 1.**
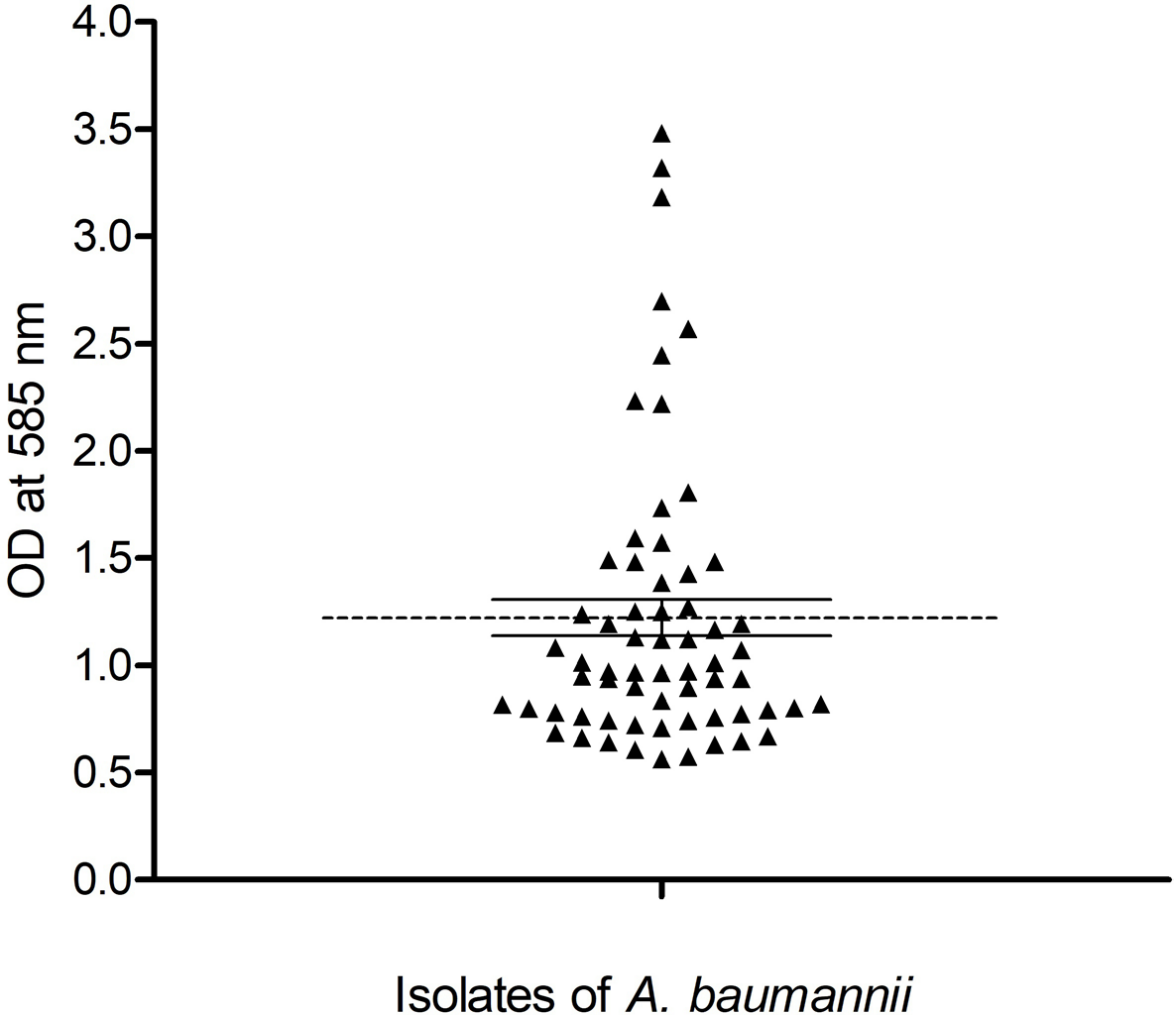
Scatter plot showing the biofilm-forming capacities of the *A. baumannii* (*n*=64) isolates as determined by crystal violet assay. Each symbol represents the average OD_585_ of triplicate wells of individual isolates. The data is presented as mean±SEM, with the dashed line indicating the mean OD_585_ of all isolates.

In this study, the biofilm-forming capacities of the isolates were assessed by crystal violet assay using a 96-well plate. The OD_585_ of the test isolates (OD_T_) ranged from 0.563 to 3.481 (Fig. 1) and the cut-off OD (OD_C_) was determined to be 0.12. The OD_T_ of all isolates were >4OD_C_ _,_ indicating that they are strong biofilm producers.

### PFGE genotyping

**Fig. 2.**
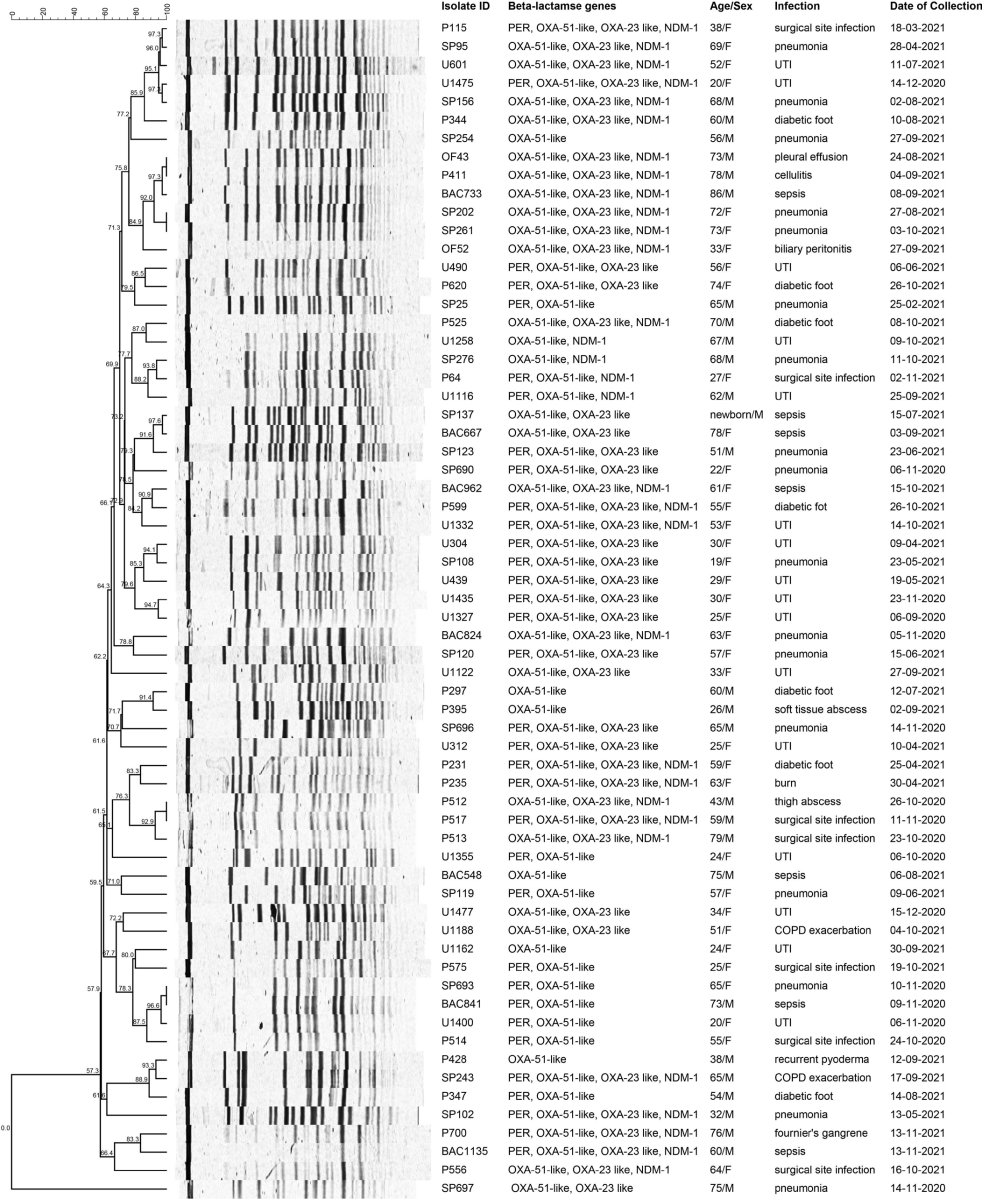
*Apa*I-PFGE dendrogram of *A. baumanni* isolates analysed in the present study.

PFGE revealed considerable heterogeneity among the isolates (Fig. 2). At 80 % similarity cut-off, 48 out of the 64 isolates formed 16 clusters, with each cluster having two or more isolates. The rest of the isolates (*n*=16) had unique banding patterns and therefore did not form part of any cluster. The two largest clusters had only six isolates each; all of them were positive for *bla*_OXA-51-like_, *bla*_OXA-23-like_ and *bla*_NDM-1_ genes. Moreover, one of these clusters contained isolates recovered in a 1 month period in 2021, indicating a possible cross-transmission. Isolates with the carbapenemase genes, *bla*_NDM-1_ and *bla*_OXA-23-like_ were distributed over 12 and 23 clusters respectively.

## Discussion

This study provides the first data on the comprehensive molecular characterization of a collection of *Acinetobacter baumannii* isolates from the southern state of Kerala, India. Kerala was the first Indian state to implement an AMR containment plan called the Kerala Antimicrobial Resistance Strategic Action Plan (KARSAP) in 2018, and the state’s first antibiogram report has recently been published [24]. In the present study, phenotypic carbapenem resistance was nearly 63 %, and multidrug-resistant (MDR) phenotype (non-susceptibility to ≥1 agent in ≥3 antimicrobial categories) was observed in 76.5 % of the isolates. Further, 36 % of the isolates belonged to the category of extensively drug-resistant (XDR) (non-susceptibility to ≥1 agent in all but ≤2 antimicrobial categories) bacteria. The classification of isolates as XDR/MDR was made according to the criteria laid down by Magiorakos *et al.* [25]. As we have analysed only a fewer number of isolates, our findings cannot be generalized and extrapolated to other settings in the region. It is noteworthy that, in our study, 53 % of the isolates from cases of pneumonia were resistant to all the antibiotics tested. In the case of isolates implicated in UTI, 56 % were resistant to eight or more antibiotics. Overall, almost 30 % of the isolates showed resistance to all the 12 antibiotics tested. A similar 1 year-long study in 2017 at a major teaching hospital in Kerala reported a carbapenem resistance of 41 % among the isolates of *Acinetobacter baumannii* implicated in lower respiratory tract infections [26]. One of the earlier studies from India which screened *A. baumannii* isolates from eight major hospitals during 2014–2017 reported 100 % resistance to imipenem and meropenem [27]. According to the National Antimicrobial Resistance Surveillance Network, imipenem resistance was 62 % among a large collection of *A. baumannii* isolates from major hospitals across India during January–December 2020 [28]. Also, more than 50 % of those isolates exhibited resistance to all the tested drugs, except minocycline to which resistance was 26 %.

All our isolates carried *bla*_OXA-51-like_, which is intrinsic to *A. baumannii* and codes for a weak carbapenemase. Carbapenem MICs for *A. baumannii* isolates which overexpress *bla*_OXA-51-like_ due to the insertion of IS*AbaI* have been found to be similar to those for isolates producing acquired carbapenemases [29]. While it is expected that the association of IS*AbaI* with *bla*_OXA-51-like_ confers resistance to carbapenems, carbapenem-susceptible isolates having IS*AbaI*/bla_OXA-51_ genotype have also been described [30]. In our study, out of the 15 isolates with *bla*_OXA-51-like_ as the sole carbapenemase determinant, only two exhibited phenotypic carbapenem resistance. However, they did not have IS*AbaI* upstream of *bla*_OXA-51-like_, suggesting the presence of non-carbapenemase mediated resistance in those isolates. The major genetic determinant of carbapenem resistance among our isolates was the *bla*_OXA-23-like_ gene followed by *bla*_NDM-1_. A similar trend has been reported among CRAB isolates in many previous studies from India [9–11, 31–33]. OXA-23 group was the first group of carbapenem-hydrolysing oxacillinases to be identified in *A. baumannii* and the genes encoding these enzymes are mostly plasmid-borne [7]. OXA-23 has disseminated worldwide and is found predominantly in isolates from the USA, India and South Korea [34]. The expression of *bla*_OXA-23-like_ is largely regulated by IS*Aba1* or IS*Aba4* found upstream of this gene by providing promoter sequences. The data on the high frequency of IS*AbaI*/*bla*_OXA-23_ structure among our isolates align with previous reports from many parts of the world including India [27, 35, 36].

The relatively higher proportion (48.4 %) of the metallo-β-lactamase gene, *bla*_NDM-1_ in the present study is a cause of concern. We also observed the co-occurrence of *bla*_NDM-1_ and *bla*_OXA-23-like_ in 27 (42.2 %) isolates; notably, these isolates showed a broad resistance profile, with 74 % of them being resistant to no less than 10 out of the 12 antibiotics tested. Isolates co-harbouring these genes have been reported sporadically from different countries including India [11, 37–40]. NDM-1 was first reported in 2008 in a *Klebsiella pneumoniae* isolate from India; however, NDM-1-positive *Acinetobacter baumannii* isolates from an Indian hospital in 2005 have been retrospectively identified as the earliest known NDM-1-carrying bacteria [41]. Since then, this gene has been acquired by various Gram-negative pathogens and has spread globally. A recent study has shown that the global dissemination of this gene is driven mainly by transposon jumps, with plasmid horizontal transfers playing more of a role in local transmission [42].

In line with the previous studies from India, the predominant ESBL gene identified among our isolates was *bla*_PER-1_, with an incidence rate of 53 % [10, 27]. However, in contrast to those studies, none of our isolates carried other ESBL genes such as *bla*_TEM_. Apparently, PER is the most common ESBL encountered in *A. baumannii* and PER-1-producing strains have been reported from many parts of the world including the USA, Europe, Asia and the Middle East [43]. Recently, it has been shown that PER-like β-lactamases contribute significantly to reduced susceptibility to cefiderocol, a novel siderophore cephalosporin with broad spectrum activity against a variety of Gram-negative pathogens [44].

Besides carbapenemase-encoding genes, various other resistance genes were also identified among our isolates. This included genes conferring resistance to fluoroquinolones (*qnrS* and *aac(6')-Ib-cr*), aminoglycosides (*strA, strB* and *aphA1-Iab*), sulphonamides (*sul1* and *sul2*) and tetracycline (*tetB*). A recent whole genome-based study on *A. baumannii* isolates (*n*=47) from India revealed the presence of as many as 79 types of ARGs of intrinsic and acquired nature [45]. Similar to our observations, high prevalence of *sul2* and *tetB* among *A. baumannii* has been reported in previous studies from India [32, 45], Pakistan [46] and Algeria [47]. Besides conferring resistance to tetracycline and doxycycline, the *tetB* gene mediates resistance to minocycline, a widely employed drug for CRAB infections with the ability to overcome most of the resistance mechanisms affecting other tetracyclines including tigecycline [48]. The streptomycin resistance genes, *strA/strB* have been frequently found in *A. baumannii* and it has been shown that a group of highly-related conjugative plasmids harbouring *sul2* and *strA*/*strB* are widely distributed in *A. baumannii* clones including the globally disseminated global clone 1 (GC1) [49]. The *aphA1-Iab* gene, also known as *aph(3')-Ic* and *aphA7* encodes an aminoglycoside-O-phosphotransferase and has sporadically been reported in *A. baumannii* isolates; however, reports are scanty on the prevalence of this gene in *Acinetobacter* species from India[50, 51]. Emergence of plasmid-mediated quinolone resistance (PMQR) is a cause of serious concern as fluoroquinolones are one of the safer and widely employed treatment options for serious Gram-negative infections. As observed for the isolates from our study, high prevalence *aac(6')-Ib-cr*, and co-occurrence of this gene with *qnrS* has been reported in *A. baumannii* isolates from a recent study at a teaching hospital in South India [52]. However, our isolates did not harbour other PMQR determinants such as *oqxAB* or *qepA* genes.

In addition to being resistant to multiple antibiotics, the isolates from this study showed reduced susceptibility to the quaternary ammonium compound (QAC), benzalkonium chloride, with 98 % of the isolates exhibiting MICs in the range of 32–128 µg ml^−1^. QACs are disinfectants with little toxicity and high microbicidal activity over a wide range of pH, and are extensively used in clinical and industrial settings [21]. Unlike antibiotics, there are no standard breakpoints determined for disinfectants to categorize the isolates as susceptible/intermediate/resistant. A study by Rajamohan *et al.* reported higher MICs (30–120 µg ml^−1^) of benzalkonium chloride for the majority of *A. baumannii* isolates analysed; however, Babaei *et al.* from Malaysia reported MICs as low as 0.02–0.2 µg ml^−1^ [53, 54]. It is noteworthy here that the in-use concentration of benzalkonium chloride in clinical settings is usually well in excess of these MICs, with disinfectant solutions containing at least 500 µg ml^−1^ of the active compound [55]. However, monitoring disinfectant and antiseptic resistance is crucial for devising infection control strategies for a pathogen like *A. baumannii* which is known to have environmental reservoirs in hospital and can infect critically ill patients.

Among the various QAC resistance genes, *qacE* and its deletion mutant *qacEΔ1,* which mediate resistance by an efflux transporter, have been frequently encountered in Gram-negative bacteria including *A. baumannii* [56]. Interestingly, all isolates from the present study carried *qacEΔ1*; previous studies have reported similar prevalence rates for this gene in *A. baumannii* [57, 58]. However, concerning the effect of *qacE*/*qacEΔ1* on the MICs of QACs, different studies have shown varying results. While studies by Babaei *et al.,* Kucken *et al.,* and Nor A’shimi *et al.* showed no correlation between the presence of *qacE*/*qacEΔ1* and increased MICs to benzalkonium chloride, Liu *et al.* reported that the carriage of *qacE* (but not *qacEΔ1*) was significantly associated with higher MIC (64 µg ml^−1^) [54, 55, 59]. Regarding chlorhexidine and triclosan, low MICs were observed among our isolates, indicating the potent antimicrobial activity of these agents. Low prevalence (~3 %) of triclosan resistance in *A. baumannii,* with the majority of isolates showing MIC of <1 µg ml^−1^, has been reported in previous studies involving large collection of isolates [60, 61].

We also assessed the biofilm-forming ability of the isolates employing the microtitre plate assay and found that all isolates belonged to the category of strong biofilm-formers. This has serious implications for infection control as biofilm-residing bacteria are often recalcitrant to antibiotic treatments and can cause persistent or recurring infections. Previous studies have shown that biofilm formation rate is higher in *A. baumannii* compared to other *Acinetobacter* species [62, 63]. Biofilm is a major virulence determinant of *A. baumannii* and is mainly regulated by the abaI/abaR quorum sensing system [64]. Also, many factors such as the biofilm-associated protein (BAP), the exopolysaccharide poly-N-acetyl glucosamine (PNAG), the extended-spectrum β-lactamse PER-1, Chaperone-Usher secretion system (CUS), and the outermembrane protein A (OmpA) have been shown to contribute to biofilm formation in *A. baumannii* [65]. However, we have not investigated the genetic basis of biofilm formation in our isolates.

The molecular subtyping of the isolates was performed by PFGE which revealed high genetic heterogeneity among the isolates (*n*=64), with no more than six isolates forming clusters at 80 % cut-off. This clearly indicates the multi-clonal nature of *A. baumannii* isolates circulated in the hospital during the study period. Apparently, the type of infection or the antibiogram of the isolates did not influence PFGE clustering; however, 50 % of the isolates from the two largest clusters were resistant to all the antibiotics tested. Also, the presence or absence of the major carbapenemase and ESBL determinants (*bla*_OXA-23_, *bla*_NDM-1_ and *bla*_PER-1_) was not associated with PFGE patterns. Nevertheless, in some instances, isolates recovered within a few days apart from each other and with identical ARG profile were found to be clustered, possibly indicating clonal dissemination of the strains. A study by Jain *et al.* has demonstrated significant cross transmission of *A. baumannii* strains between the patients and the ICU environment at an Indian hospital by employing PFGE [66]. *Apa*I-PFGE is considered the gold standard for studying the local epidemiology of *A. baumannii* and has been widely used in outbreak investigations [67]. However, the discriminatory power of PFGE to accurately distinguish *A. baumannii* isolates with close genetic backgrounds has been found low, with other approaches such as multi-locus sequence typing (MLST_oxford) and core-genome MLST showing better resolution capabilities [68].

In conclusion, this study demonstrates a diverse population of clinical *A. baumannii* from this region*,* with *bla*_OXA-23_ and *bla*_NDM-1_ as major carbapenemase determinants. The high proportion of isolates exhibiting MDR phenotype, reduced disinfectant susceptibility and strong biofilm-forming ability is a cause for concern. Continuous surveillance and stringent infection control measures are key to manage this pathogen in nosocomial settings.

## Supplementary Data

Supplementary material 1
