# Supplementary material for: Genotyping of Acinetobacter baumannii isolates from a tertiary care hospital in Cochin, South India
Source: Access Microbiol. 2023 Nov 28;5(11):000662.v4. doi: 10.1099/acmi.0.000662.v4 (PMC10702374; doi:10.1099/acmi.0.000662.v4)
Supplement: Supplementary material 1 [file acmi-5-662.v4-s001.pdf]

Supplementary Table 1: Details of the primers used in this study for screening antibiotic/disinfectant resistance genes

Primer	Primer sequence (5'-3')	Target gene	Amplicon size	Annealing Temperature	Reference
	**Detection of antibiotic resistance genes**				
**Multiplex primers**					
OXA-23-likeF	GATCGGATTGGAGAACCAGA	$bla_{OXA\text{-}23\text{-}like}$	501 bp	52 °C	Woodford et al., 2006
OXA-23-likeR	ATTTCTGACCGCATTTCCAT				
OXA-24-likeF	GGTTAGTTGGCCCCCTTAAA	$bla_{OXA\text{-}24\text{-}like}$	246 bp		
OXA-24-likeR	AGTTGAGCGAAAAGGGGATT				
OXA-51-likeF	TAATGCTTTGATCGGCCTTG	$bla_{OXA\text{-}51\text{-}like}$	353 bp		
OXA-51-likeR	TGGATTGCACTTCATCTTGG				
OXA-58-likeF	AAGTATTGGGGCTTGTGCTG	$bla_{OXA\text{-}58\text{-}like}$	599 bp		
OXA-58-likeR	CCCCTCTGCGCTCTACATAC				
ISAbaI-F	CACGAATGCAGAAGTTG	IS*AbaI*	549 bp	56 °C	Segal et al., 2005
ISAbaI-R	CGACGAATACTATGACAC				
**Multiplex primers**					
MultiGES_for	AGTCGGCTAGACCGGAAAG	GES-1 to 9 and GES-11	399 bp	60 °C	Dallenne et al., 2010
MultiGES_rev	TTTGTCCGTGCTCAGGAT				
MultiPER_for	GCTCCGATAATGAAAGCGT	PER-1 and PER-3	520 bp		
MultiPER_rev	TTCGGCTTGACTCGGCTGA				
MultiVEB_for	CATTTCCCGATGCAAAGCGT	VEB-1 to VEB-6	648 bp		
MultiVEB_rev	CGAAGTTTCTTTGGACTCTG				
**Multiplex primers**					
MultiIMP_for	TTGACACTCCATTTACDG	IMP variants except IMP-9, IMP-16,	139 bp	55 °C	
MultiIMP_rev	GATYGAGAATTAAGCCACYCT	IMP-18, IMP-22 and IMP-25			
MultiVIM_for	GATGGTGTTTGGTCGCATA	VIM variants including VIM-1 and VIM-2	390 bp		
MultiVIM_rev	CGAATGCGCAGCACCAG				
MultiKPC_for	CATTCAAGGGCTTTCTTGCTGC		538 bp		
MultiKPC_rev	ACGACGGCATAGTCATTTGC	KPC-1 to KPC-5			
**Multiplex primers**					
MultiCTXMGp1_for	TTAGGAARTGTGCCGCTGYA	variants of CTX-M group 1 including	688 bp	60 °C	
MultiCTXMGp1_rev	CGATATCGTTGGTGGTRCCAT	CTX-M-1, CTX-M-3 and CTX-M-15			
MultiCTXMGp2_for	CGTTAACGGCACGATGAC	variants of CTX-M group 2 including	404 bp		
MultiCTXMGp2_rev	CGATATCGTTGGTGGTRCCAT	CTX-M-2			
MultiCTXMGp9_for	TCAAGCCTGCCGATCTGGT	variants of CTX-M group 9 including	561 bp		
MultiCTXMGp9_rev	TGATTCTCGCCGCTGAAG	CTX-M-9 and CTX-M-14			

**Multiplex primers**<br>MultiTSO-T_for<br>MultiTSO-T_rev<br>MultiTSO-S_for<br>MultiTSO-S_rev<br>MultiTSO-O_for<br>MultiTSO-O_rev	 CATTTCCGTGTCGCCCTTATTC<br>CGTTCATCCATAGTTGCCTGAC<br>AGCCGCTTGAGCAAATTAAAC<br>ATCCCGCAGATAAATCACCAC<br>GGCACCAGATTCAACTTTCAAG<br>GACCCCAAGTTTCCTGTAAGTG	 TEM variants including TEM-1 and TEM-2<br>SHV variants including SHV-1<br><br>OXA-1, OXA-4 and OXA-30	 800 bp<br><br>713 bp<br><br>564 bp	60 °C	
NDM-1_a_fw<br>NDM-1_a_rev	CAATATTATGCACCCGGTCG<br>CCTTGCTGTCCTTGATCAGG	*bla*$_{NDM-1}$	632 bp	52 °C	Kaase et al., 2012
qnrA-F<br>qnrA-R	ATTTCTCACGCCAGGATTTG<br>GATCGGCAAAGGTTAGGTCA	*qnrA*	516 bp	56 °C	Sivaraman et al., 2021
qnrB-F<br>qnrB-R	GATCGTGAAAGCCAGAAAGG<br>ATGAGCAACGATGCCTGGTA	*qnrB*	476 bp		
qnrS-F<br>qnrS-R	GCAAGTTCATTGAACAGGGT<br>TCTAAACCGTCGAGTTCGGCG	*qnrS*	428 bp		
qepA-F<br>qepA-R	CGTGTTGCTGGAGTTCTTC<br>CTGCAGGTACTGCGTCATG	*qepA*	403 bp	57 °C	
oqxA-F<br>oqxA-R	GACAGCGTCGCACAGAATG<br>GGAGACGAGGTTGGTATGGA	*oqxA*	339 bp	54 °C	
oqxB-F<br>oqxB-R	CGAAGAAAGACCTCCCTACCC<br>CGCCGCCAATGAGATACA	*oqxB*	240 bp		
aac(6')-Ib-cr-F<br>aac(6')-Ib-cr-R	TTGCGATGCTCTATGAGTGGCTA<br>CTCGAATGCCTGGCGTGTTT	*aac(6')-Ib-cr*	482 bp	55 °C	
strA-F<br>strA-R	CCTGGTGATAACGGCAATTC<br>CCAATCGCAGATAGAAGGC	*strA*	546 bp		
strB-F<br>strB-R	ATCGTCAAGGGATTGAAACC<br>GGATCGTAGAACATATTGGC	*strB*	509 bp		
aphA1-Iab-F<br>aphA1-Iab-R	AAACGTCTTGCTCGAGGC<br>CAAACCGTTATTCATTCGTGA	*aphA1-Iab*	461 bp	56 °C	Wajid et al., 2018
sul1-F<br>sul1-R	CGGCGTGGGCTACCTGAACG<br>GCCGATCGCGTGAAGTTCCG	*sul1*	433 bp	67 °C	Sivaraman et al., 2021
sul2-F<br>sul2-R	GCGCTCAAGGCAGATGGCATT<br>GCGTTTGATACCGGCACCCGT	*sul2*	293 bp		
tetA-F<br>tetA-R	GGTTCACTCGAACGACGTCA<br>CTGTCCGACAAGTTGCATGA	*tetA*	577 bp	56 °C	
tetB-F<br>tetB-R	CCTCAGCTTCTCAACGCGTG<br>GCACCTTGCTGATGACTCTT	*tetB*	634 bp		
	**Detection of disinfectant resistance genes**				
qacEΔ1-F	AATCCATCCCTGTCGGTGTT	*qacEΔ1*	175 bp	56 °C	

qacEΔ1-R	CGCAGCGACTTCCACGATGGGGAT				
qacE-F<br>qacE-R	AAGTAATCGCAACATCCG<br>CTACTACACCACTAACTATGAG	*qacE*	258 bp	50 °C	Zou et al., 2014
qacF-F<br>qacF-R	GTCGTCGCAACTTCCGCACTG<br>TGCCAACGAACGCCCACA	*qacF/H/I*	229 bp	60 °C	
qacG-F<br>qacG-R	TCGCCTACGCAGTTTGGT<br>AACGCCGCTGATAATGAA	*qacG*	122 bp	56 °C	
